# Catanionic solid lipid nanoparticles with surface 5-HT-moduline are efficacious nanocarriers to target endothelial cells for potential cardiac imaging

**DOI:** 10.1186/1532-429X-17-S1-P250

**Published:** 2015-02-03

**Authors:** Yung-Chih Kuo

**Affiliations:** National Chung Cheng University, Chia-Yi, Taiwan

## Background

This study investigates the transcytotic capability of 5-HT-moduline-grafted catanionic solid lipid nanoparticles (CASLNs) to human endothelia.

## Methods

5-HT-moduline is crosslinked onto CASLNs and 5-HT-moduline-modified CASLNs (5-HT-moduline/CASLNs) are administered to traverse an endothelial monolayer.

## Results

CASLNs were prepared in catanionic microemulsion and constructed into solid colloids by rapid cooling. In addition, the uptake of 5-HT-moduline/CASLNs by human endothelia was visualized by immunochemical staining. We found that an increase in the concentration of catanionic surfactants reduced the viability of endothelia. Moreover, an increase in the concentration of 5-HT-moduline reduced the grafting efficiency of 5-HT-moduline, cell viability, and transendothelial electrical resistance, and enhanced the permeability of propidium iodide. Although 5-HT-moduline/CASLNs may jeopardize the endothelial viability and add complexity of preparation, their efficiency in the targeting delivery to endothelia is significantly higher than CASLNs.

## Conclusions

5-HT-moduline/CASLNs can be promising delivery nanocarriers to transport sensing reagents to endothelia and of potential as a cardiac visualizing system.

## Funding

This work is supported by the Ministry of Science and Technology of the Republic of China.

